# IL-15 and a Two-Step Maturation Process Improve Bone Marrow-Derived Dendritic Cell Cancer Vaccine

**DOI:** 10.3390/cancers11010040

**Published:** 2019-01-04

**Authors:** Ananda Mookerjee, Michele Graciotti, Lana E. Kandalaft

**Affiliations:** 1 Ovarian Cancer Research Center, University of Pennsylvania, Philadelphia, PA 19104, USA; mookerjeeananda@gmail.com or ananda.mookerjee@mssm.edu; 2 Ludwig Cancer Research Center, University of Lausanne, Épalinges 1066, Switzerland; michele.graciotti@chuv.ch; 3 Department of Oncology, University Hospital of Lausanne, Lausanne 1011, Switzerland

**Keywords:** cancer vaccine, dendritic cell, ovarian cancer, interleukin 15, squaric acid

## Abstract

In the last 20 years, dendritic cells (DCs) have been largely used as a platform for therapeutic vaccination in cancer patients. However, despite its proven safety and ability to induce cancer specific immune responses, the clinical benefits of DC-based immunotherapy are currently very limited. Thus, novel approaches are still needed to boost its efficacy. Our group recently showed that squaric acid treatment of antigens is an important adjuvant that can increase vaccine-induced downstream immune responses and therapeutic outcomes. Here we further improved this dendritic cell vaccine formulation by developing a new method for differentiating and maturing DCs from their bone marrow precursors. Our data demonstrate that bone marrow-derived DCs differentiated with GM-CSF and IL-15 and matured with a maturation cocktail in two steps present a more mature and immunogenic phenotype, compared to standard DC preparations. Further suppression of the prostaglandin E_2_ pathway achieved even more immunogenic DC phenotypes. This vaccine was more potent at delaying tumor growth, improved animal survival and induced a more immunogenic and Th1-skewed T cell response in an ovarian cancer mouse model. These promising results support future efforts for the clinical translation of this approach.

## 1. Introduction

Ovarian cancer is one of the most severe gynecologic cancers and has a very high mortality rate. Over 230,000 women are diagnosed with ovarian cancer worldwide each year, and about 140,000 women die from the disease [1]. Unfortunately, 85% of all ovarian cancer cases are detected only at a late-stage, with a 5-year survival rate of 39% [1].

Previous evidence demonstrated that despite the relatively low mutational burden in ovarian carcinoma compared to other cancer types [2,3], tumor infiltrating T lymphocytes naturally occur in >50% of ovarian cancer patients, a feature that well correlates with improved clinical outcomes [4]. Furthermore, T cells isolated from ovarian cancer patients are able to recognize tumor-associated antigens (TAAs) and exhibit tumor-specific cytotoxic activity in vitro [5]. Based on this collective evidence, subsequent clinical studies employed dendritic cell (DC) based cancer vaccines in an attempt to stimulate and sustain a tumor specific T cell response. These studies [6] (and others for other types of cancer) [7] importantly demonstrated the clinical safety and feasibility of DC based vaccines. However, despite the crucial role of DCs in stimulating and orchestrating both innate and adoptive immune responses [8], the objective response rates of clinical studies with DC cancer vaccines rarely exceeded 15% [9,10]. Hence, it is generally believed that we have not yet harnessed the true potential of DC-based anti-cancer vaccines and novel strategies should be developed to improve current therapeutic outcomes [11].

Two aspects of DC immunobiology are particularly crucial in designing a DC vaccine: the antigen source and the differentiation and maturation stimuli engaged. Our group previously focused on optimizing whole tumor cell lysate (WTL) preparation identifying in particular HOCl [12,13] and squaric acid [14] as two potent treatments able to increase the immunogenicity of the WTL antigen source. In this study we focused instead on the optimization of the differentiation and maturation stimuli applied respectively to DC precursors and immature DCs in order to reach their fully immunogenic potential. The most widely accepted protocol to prepare DCs for clinical cancer vaccination involves the differentiation of DCs from their isolated precursors (peripheral blood monocytes in humans or bone marrow cells in mice) in the presence of granulocyte-macrophage colony-stimulating factor (GM-CSF) and interleukin-4 (IL-4) [15]. In more recent years, an alternative type of DCs called “IL-15 DCs” differentiated in the presence of GM-CSF and interleukin-15 (IL-15) has also emerged as an efficient and potentially more immunogenic vaccine for therapeutic applications [16,17,18]. Instead, for maturation, a much wider variety of cytokine cocktails have been used by different groups [10]. In particular, a cocktail mixture containing tumor necrosis factor-α (TNF-α), interleukin-1β (IL-1β), interleukin-6 (IL-6), and prostaglandin E_2_ (PGE2), is currently considered the gold standard for DC maturation and had been largely used in the context of anti-cancer therapeutic vaccines [19,20]. This mix is able to efficiently induce expression of common DC surface maturation markers, uniform DC maturation, as well as high levels of T cell proliferation and priming [19]. However, DCs matured with this mixture failed to produce significant levels of interleukin-12 (IL-12) a crucial signal for T cell activation and Th1 differentiation [21]. On the other hand, our group previously demonstrated that DC maturation in the presence of lipopolysaccharide (LPS) and interferon-γ (IFN-γ) led to high levels of IL-12 production [22,23], an approach that was subsequently translated also into a phase I clinical trial with promising results [12,13,24]. In addition to this, other groups also successfully matured DCs in the presence of CD40 ligand and IFN-γ [25], or toll-like receptor ligands [26].

**Figure 1:**
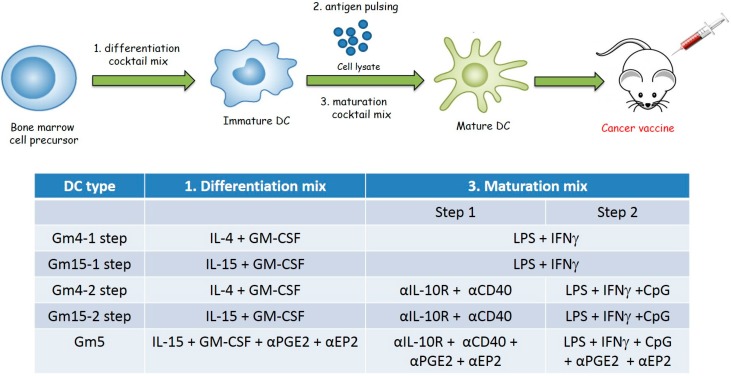
Schematic representation of the DC preparation protocol for animal vaccination. DC cell precursors were first isolated from the bone marrow of tumor-bearing mice and subsequently incubated with the stimuli indicated in the table for 4 days (1. differentiation mix). Immature DCs were then pulsed with squaric acid-treated ID8 cell lysate by overnight incubation. Finally, DCs were then matured by incubation with the stimuli indicated in the table (3. maturation mix). In particular, the maturation process was achieved either in one single step with 24 h incubation, or in two subsequent steps with incubation with the indicated reagents for 3 h (step 1), followed by overnight incubation with the indicated reagents (step 2). (DC: dendritic cells)

Based on this collective evidence, in this study, we characterize and compare the efficacy of DCs differentiated with either IL-4 or IL-15 and a cocktail of differentiation and maturation stimuli in a mouse model of metastatic ovarian cancer in an attempt to improve currently available DC vaccine formulations (Figure 1). Our results demonstrate that DCs differentiated with GM-CSF and IL-15 and matured in the presence of anti-CD40, anti-PGE2, anti-PGE2 receptor (EP2), anti-IL-10 receptor antibodies and CpG oligonucleotides (CpG) (in addition to the canonical LPS and IFNγ) exhibited higher levels of anti-tumor response compared to canonical DC formulations. These results strongly support further implementation of this DC formulation in future cancer vaccine clinical trials.

## 2. Results

### 2.1. Differentiation with GM-CSF and IL-15 Yields a More Immunogenic DC Phenotype than Canonical DCs Differentiated with GM-CSF and IL-4

**Figure 2:**
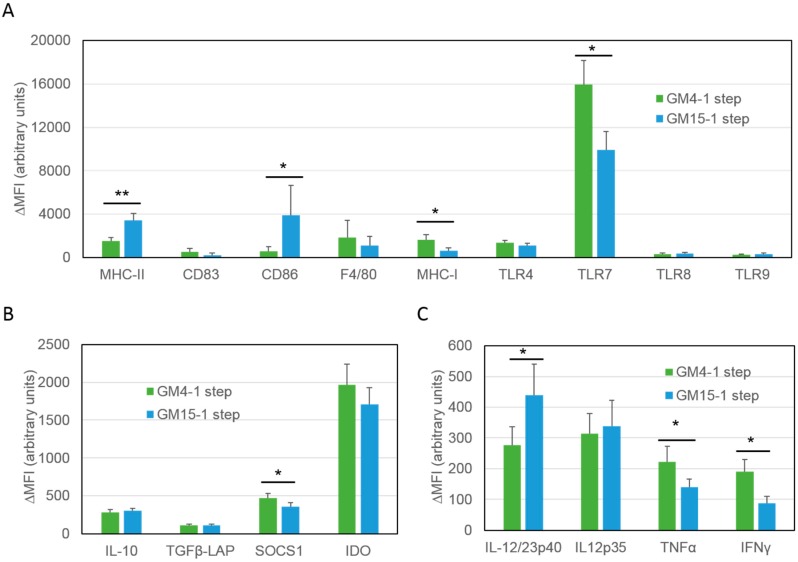
Phenotypic comparison between mouse DCs differentiated ex vivo from bone marrow precursors in the presence of GM-CSF and either IL-4 or IL-15. (**A**–**C**) Isolated mouse bone marrow cells were differentiated in vitro for 4 days in the presence of GM-CSF and either IL-4 (GM4-1 step) or IL-15 (GM15-1 step) as indicated, pulsed with an ID8 tumor cell lysate treated with squaric acid and subsequently matured with LPS/IFNγ. Expression levels of indicated markers were then assessed by antibody staining followed by FACS analysis. The net mean fluorescence intensity (ΔMFI = Raw MFI-MFI of Isotype) for each marker is reported in the graph; data are representative of 3 independent experiments. Significant differences were assessed with unpaired Student’s t test and indicated with asterisks: * *p* < 0.05; ** *p* < 0.01. (DC: dendritic cells; GM-CSF: granulocyte-macrophage colony-stimulating factor; IL-4: interleukin-4; IL-15: interleukin-15; IFN-γ: interferon-γ; LPS: lipopolysaccharide).

In order to compare their immunogenic phenotype, we first differentiated DCs in vitro from the bone marrow of tumor bearing animals with GM-CSF and IL-4 (GM4-1 step DCs) or with GM-CSF and IL-15 (GM15-1 step DCs), pulsed with a squaric acid treated-ID8 tumor cell lysate (LSQ) and finally matured them with LPS plus IFNγ. After cell maturation, DC phenotypes were assessed by antibody staining against common surface markers such as: MHC-I, MHC-II, CD83, CD86, F4/80, and Toll-like receptors (TLRs) (Figure 2A, Figure S1) as well as for intracellular levels of key immunosuppressive (Figure 2B) and immunostimulatory (Figure 2C) cytokines by FACS analysis. Results showed that GM15-1 step DCs have significantly much higher MHC-II and CD86 expression, but lower MHC-I and TLR7 expression compared to GM4-1 step DCs (Figure 2A). In terms of cytokine release, although GM15-1 step DCs produced significantly lower TNFα and IFNγ, these DCs generated more IL-12/23p40 and less SOCS1 compared to GM4-1 step DCs. Other tested markers such as interleukin 10 (IL-10), TLR4, TLR8, transforming growth factor-β (TGFβ) and indoleamine 2,3-dioxygenase (IDO) did not reveal any significant changes (Figure 2A–C).

Given the advantageous increase in IL-12 as well as class-II MHC and CD86, and recent data suggesting the improved efficacy DCs differentiated with IL-15 [16,27,28,29], we chose the GM15-1 step backbone for further improvements, focusing in particular on enhancing IFNγ production.

**Figure 3:**
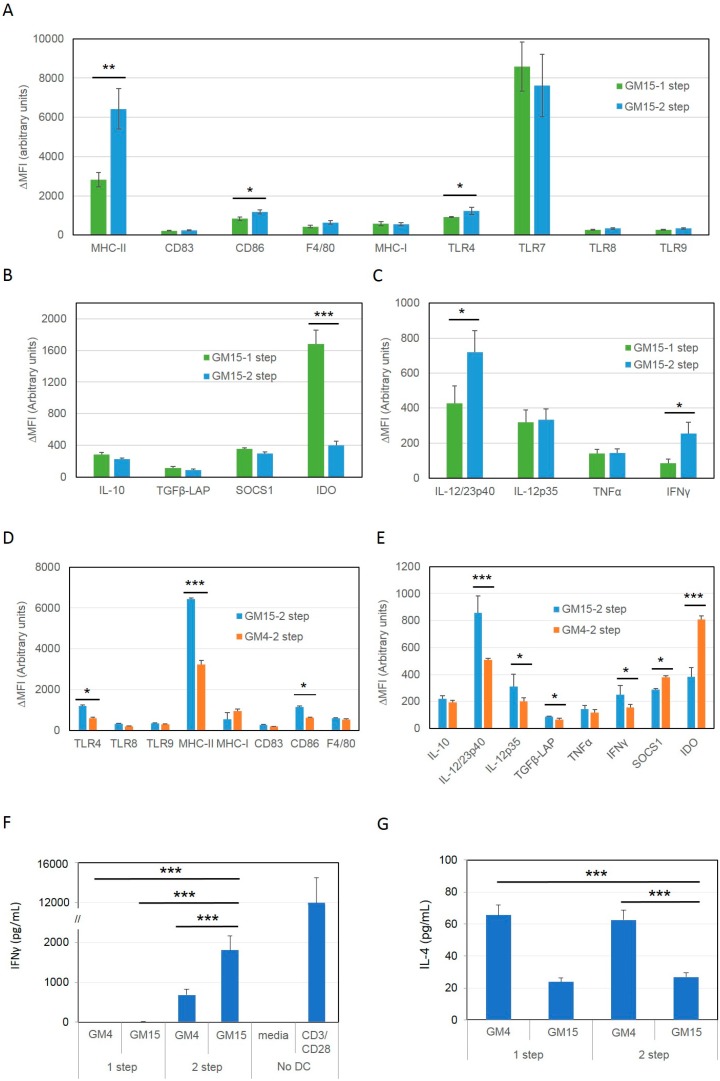
DCs matured with a two-step protocol in the presence of anti-CD40 and anti-IL10R antibodies for 24 h, followed by LPS/IFNγ/CpG stimuli present a more mature phenotype and stimulate a more Th1-skewed T cell response compared to canonical LPS/IFNγ maturation. (**A**–**E**) Immature antigen-pulsed DCs were obtained in the presence of either IL4 (GM4) or IL-15 (GM15) as reported in Figure 2. Cells were then pulsed with an ID8 tumor cell lysate treated with squaric acid and subsequently matured in the presence of either LPS/IFNγ for 24 h (GM15-1 step, GM4-1 step) or with a cocktail mix containing anti-CD40 and anti-IL-10R for 24 h, followed by a second mix containing LPS, IFNγ and CpG for the subsequent 24hr (GM4-2 step, GM15-2 step). Expression levels of indicated markers were then assessed by antibody staining followed by FACS analysis. The net mean fluorescence intensity (ΔMFI = Raw MFI-MFI of Isotype) for each marker is reported in the graph. (**F**,**G**) IFNγ and IL-4 production measured by ELISA after 24 h co-culturing of splenic T-lymphocytes isolated from tumor bearing animals with the indicated DC formulations. Data are representative of at least 3 independent experiments. Significant differences were assessed with unpaired Student’s t test and indicated with asterisks: * *p* < 0.05; ** *p* < 0.01; *** *p* < 0.005. (CpG: CpG oligonucleotides; DC: dendritic cells; GM-CSF: granulocyte-macrophage colony-stimulating factor; IL-4: interleukin-4; IL-10R: interleukin-10 receptor; IL-15: interleukin-15; IFN-γ: interferon-γ; LPS: lipopolysaccharide).

In particular, based on previous evidence showing the ability of CD40 ligand to induce DC maturation and activation [30,31,32] and the ability of stimulated DCs to produce high levels of IL-10 [33,34], we decided to include both anti-CD40 and IL-10 receptor (IL-10R) antibodies in an attempt to improve the maturation process and achieve a more immunogenic DC phenotype. To achieve this, after differentiation with GM-CSF and IL-15 and LSQ antigen pulsing we applied the maturation stimuli in two steps, incubating DCs first with anti-CD40 plus anti-IL10R antibodies for 24 h, followed by the well-established maturation cocktail containing LPS and IFNγ with the addition of CpG (a potent TLR agonist [35]), for the subsequent 24 h (GM15-2 step DCs). After maturation we assessed the phenotype of these DCs by FACS analysis (Figure 3A–E).

Interestingly, introducing this new maturation scheme in DCs differentiated with GM-CSF and IL-15 (GM15-2 step DCs) led to a further and significant increase in MHC-II, CD86 as well as TLR4, and decrease in IDO expression, compared to IL-15 DCs matured in the presence of just LPS and IFNγ (GM15-1 step DCs, Figure 3A,B). A significant increase in IL-12/23p40 and IFNγ production was seen in GM15-2 step DCs compared to GM15-1 step DCs (Figure 3C).

In addition to this, we then further compared the same 2-step maturation protocol in DCs differentiated in the presence of IL-4/GM-CSF (GM4-2 step DCs), or IL-15/GM-CSF. Our results showed that the latter (GM15-2 step DCs) exhibited higher MHC-II, CD86, TLR4, IL-12/23p40, IL-12p35 and IFNγ, and lower IDO and suppressor of cytokine signaling 1 (SOCS1) relative to GM4-2 step DCs, suggesting a potentially more immunogenic phenotype (Figure 3D,E).

Next, we proceeded to compare the efficiency of these different DC formulations in eliciting anti-tumor T cell responses. To achieve this, we prepared DCs as above, we incubated them with purified T cells from the spleen of ID8 tumor bearing mice for 24 h, and finally measured cytokines levels in the supernatant by ELISA. Results showed that GM15-2 step DCs induced significantly higher IFNγ production compared to all the other tested DC formulations and lower IL-4 generation compared to IL-4 derived DCs, both trends indicative of a Th1-skewed T cell response (Figure 3F,G). Similar results were also obtained from T cells purified from mesenteric and inguinal draining lymph nodes or peritoneum (Figure S2).

**Figure 4:**
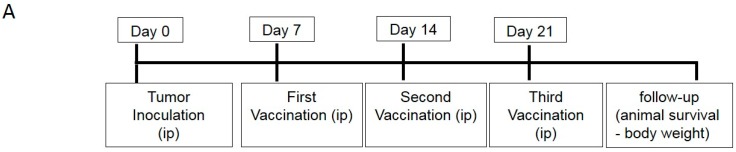

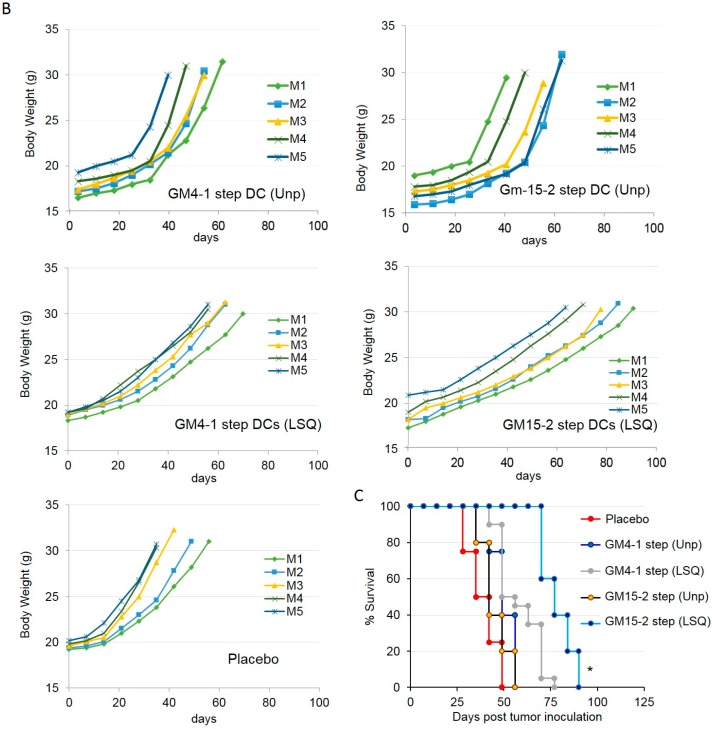
DCs matured with a two-step protocol in the presence of anti-CD40 and anti-IL10R antibodies for 24 h, followed by LPS/IFNγ/CpG stimuli prolong animal survival in a vaccination mouse model of ovarian cancer. (**A**) Vaccination scheme: mice were inoculated i.p. with ID8 cells at day 0, followed by i.p. vaccination with the indicated DC formulations (pulsed with an ID8 tumor cell lysate treated with squaric acid (LSQ) or kept unpulsed (Unp)) at day 7, 14 and 21 (*n* ≥ 5 for each group). (**B**,**C**) Body weight and animal survival were evaluated and plotted in Kaplan–Meier cumulative survival plots. Student’s t test results (comparing GM15-2 step DCs (LSQ) to both GM4-1 step DCs (LSQ) and placebo) is indicated with an asterisk: * *p* < 0.05. (CpG: CpG oligonucleotides; DC: dendritic cells; IFN-γ: interferon-γ; LPS: lipopolysaccharide).

Following in vitro studies, we proceeded to compare the efficacy of GM15-2 step DCs with the conventional GM4-1 step DCs in vivo in the ID8 ovarian cancer mouse model. To achieve this, we first inoculated ID8 cells intraperitoneally (i.p.) in C57BL/6 mice on day 0 and subsequently injected GM4-1 step or GM15-2 step DCs or placebo (phosphate-buffered saline (PBS)) i.p. on day 7, day 14 and day 21 post tumor inoculation, then following animal survival over time (Figure 4A). Interestingly, we observed that mice vaccinated with GM15-2 step DCs displayed a significant distinct survival advantage over mice vaccinated with conventional DCs (GM4-1 step DCs; *p* < 0.05) or placebo (*p* < 0.05) (Figure 4B). In particular, the median survival in the placebo group was ~40 days, while mice receiving GM4-1 step DCs had a median survival of about ~65 days, compared to ~80 days for the GM15-2 step DC-treated group. These results confirmed therefore the superiority of GM15-2 step DCs also in the in vivo context compared to canonical IL-4 differentiated DCs (GM4-1 step DCs).

### 2.2. Improving the Efficacy of GM15-2 Step DCs through Inhibiting the Prostaglandin Pathway

**Figure 5:**
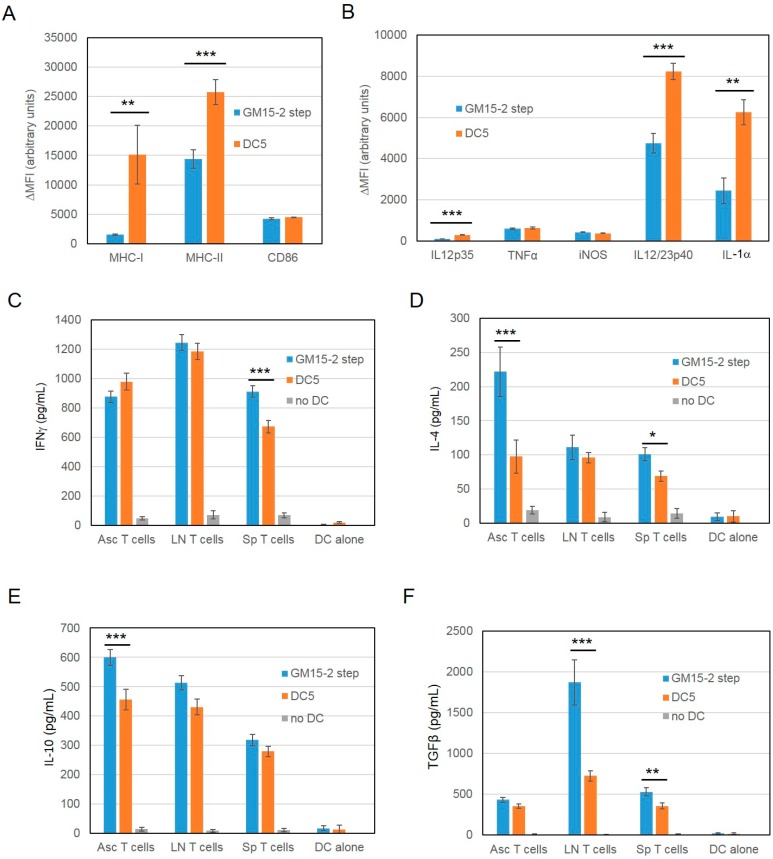
Blocking the prostaglandin E_2_ pathway during DC differentiation and maturation improves DC phenotype and in vitro immunogenicity. (**A**,**B**) Isolated mouse bone marrow cells were differentiated in the presence of GM-CSF and IL-15 (GM15-2 step DCs) for 4 days or first with GM-CSF and IL-5 for 24 h then supplemented with anti-PGE2, anti-EP2 Ab (DC5); cells were then pulsed and matured as reported in Figure 3 for GM15-2 step DCs with the addition of anti-PGE2, anti-EP2 Ab for DC5 throughout the cell culturing period. Cell phenotype was then analyzed by FACS staining and the net mean fluorescence intensity (ΔMFI = Raw MFI-MFI of Isotype) for each marker was reported in the graph. (**C**–**F**) Levels of indicated cytokines measured by ELISA, produced after 24 h co-culturing of T-lymphocytes isolated from the ascites (Asc), lymph node (LN), or spleen (Sp) of tumor bearing animals with the indicated DC formulations pulsed with an ID8 tumor cell lysate treated with squaric acid. Data are representative of at least 3 independent experiments. Significant differences were assessed with unpaired Student’s t test and indicated with asterisks: * *p* < 0.05; ** *p* < 0.01; *** *p* < 0.005. (CpG: CpG oligonucleotides; DC: dendritic cells; EP2: PGE2 receptor; GM-CSF: granulocyte-macrophage colony-stimulating factor; IL-15: interleukin-15; PGE2: prostaglandin E2).

Based on previous evidence suggesting an immunosuppressive activity of PGE2 on DC differentiation [36], function [37,38] and IL-12 production [36], we next focused on further improving our DC preparation protocol to achieve an even more immunogenic DC vaccine by introducing an antibody against PGE2 and one against its putative receptor EP2 throughout the DC culture. The phenotype of the resultant DCs (DC5) was then assessed by antibody staining followed by FACS analysis (Figure 5A,B). Results showed significantly increased levels of MHC-I, MHC-II, IL-12p35, IL12/23p40 and IL-1α in DC5, compared to the previously developed GM15-2 step DCs, while CD86, TNFα and cytokine-inducible nitric oxide synthase (iNOS) levels were essentially comparable (Figure 5A,B).

We then tested the in vitro induced T-cell responses by co-culturing GM15-2 step DCs or DC5 (prepared from bone marrow cells of tumor bearing animals) with T cells derived from ascites, spleen or draining lymph nodes of tumor bearing animals for 24 h and subsequently measuring levels of IFNγ, IL-4, TGFβ and IL-10 in the culture supernatant by ELISA. Results showed that while levels of IFNγ were essentially comparable between the two different DC preparations in use (when comparing between same T cells types, Figure 5C; except in the case of splenic T cells), IL-4 and IL-10 levels were significantly lower in co-cultures of ascites T cells with LSQ antigen-pulsed DC5 DCs compared to the respective GM15-2 step DC counterpart (Figure 5D,E). DC5 DCs also induced significantly less IL-4 when co-cultured with splenic T cells compared to GM15-2 step DCs (Figure 5D). Finally, LSQ antigen-pulsed DC5 elicited significantly lower amounts of TGFβ in co-cultures of lymph node and splenic T cells compared to GM15-2 step DCs (Figure 5F). Thus, the in vitro results here presented, taken collectively, suggest that DC5 display a more advantageous DC phenotype than the previously characterized GM15-2 step DCs, they induce a more favorable cytokine profile in co-cultured T cells and they can be therefore considered more immunogenic.

**Figure 6:**
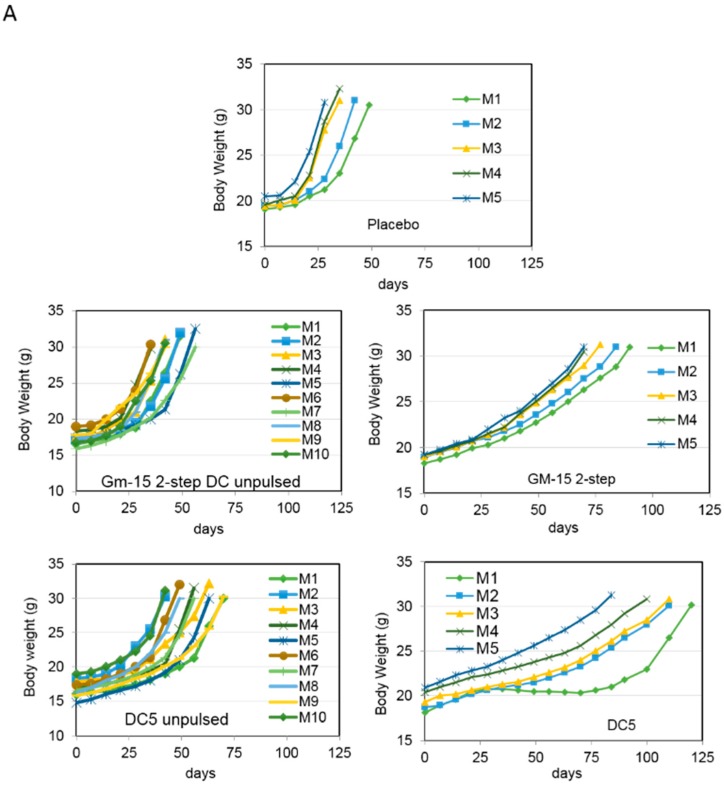

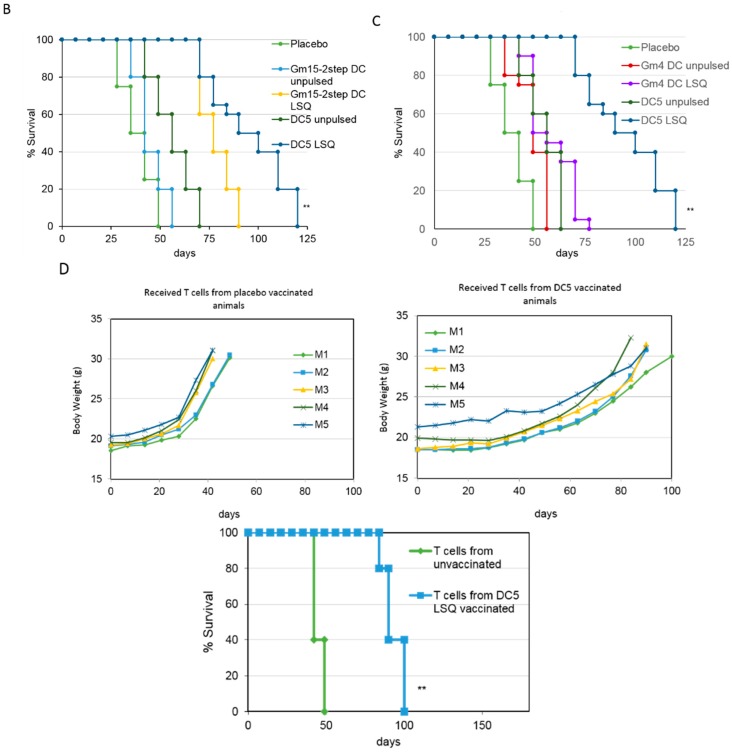
Blocking the prostaglandin E_2_ pathway during DC differentiation and maturation prolongs animal survival in a vaccination mouse model of ovarian cancer. (**A**–**C**) Mice were inoculated i.p. with ID8-fast cells at day 0, followed by i.p. vaccination with the indicated DC formulations at day 7, 14 and 21 (*n* ≥ 5 for each group). Body weight and animal survival were evaluated and plotted in Kaplan–Meier cumulative survival plots. Student’s t test results (comparing DC5 LSQ DCs to both GM15-2 step LSQ and classical GM4-1 step LSQ DCs) are indicated with an asterisk: ** *p* < 0.01 (**D**) Animals were vaccinated as reported above; T cells were then isolated from inguinal LN on day 28 post tumor inoculation and injected in the tail vein of recipient mice 3 days before tumor challenge with i.p. injection of ID8 fast cells. Body weight and animal survival were evaluated and plotted in Kaplan–Meier cumulative survival plots. Student’s t test results are indicated with an asterisk: ** *p* < 0.01. (DC: dendritic cells).

We next proceeded to test the therapeutic efficacy of DC5 in vivo. To achieve this, we injected i.p. 8–10 week old female mice with ID8 cells on day 0. We then injected i.p. placebo (PBS), GM15-2 step or DC5 unpulsed or LSQ antigen-pulsed on days 7, 14 and 21 post tumor inoculation and followed animal survival over time. We observed that therapeutic vaccination with antigen-pulsed DC5 could significantly (*p* < 0.01) delay tumor progression and impart a much higher survival advantage compared to vaccination with LSQ antigen-pulsed GM15-2 step DCs or classical GM4-1 step DCs (Figure 6A–C). While the median survival for placebo controls was 36 days post inoculation, this increased to 49 days with therapeutic vaccination with LSQ antigen-pulsed GM4 DCs, to 65 days with GM15-2 step DCs and to 110 days with DC5 DCs.

To test the tumor specificity of the T cell response as well as the memory response elicited by therapeutic vaccination with DC5 DCs, we then performed adoptive T cell transfer experiments. The donors were ID8 tumor bearing female C57BL/6 mice, either kept untreated or therapeutically treated with DC5 (i.p.) on days 7, 15 and 21 post tumor inoculation. Lymph node (LN) cells were then collected from inguinal LN on day 28 post tumor inoculation and T cells isolated by negative selection. T cells were then injected in the tail vein of recipient mice 3 days before tumor challenge with i.p. injection of ID8 cells. Animal survival measurements presented in Figure 6D showed that T cells from lymph nodes of animals therapeutically vaccinated with DC5, when transferred to naïve animals, conferred considerable prophylactic protection and delayed tumor establishment and progression compared to that conferred by T cells from placebo controls.

### 2.3. Characterization of the T Cell Response Induced by Vaccination with DC5 DCs

**Figure 7:**
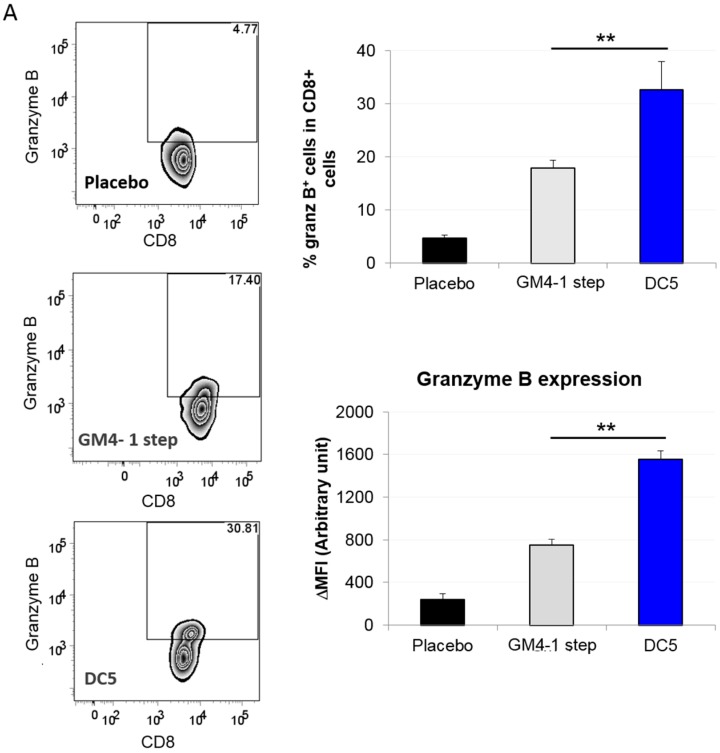

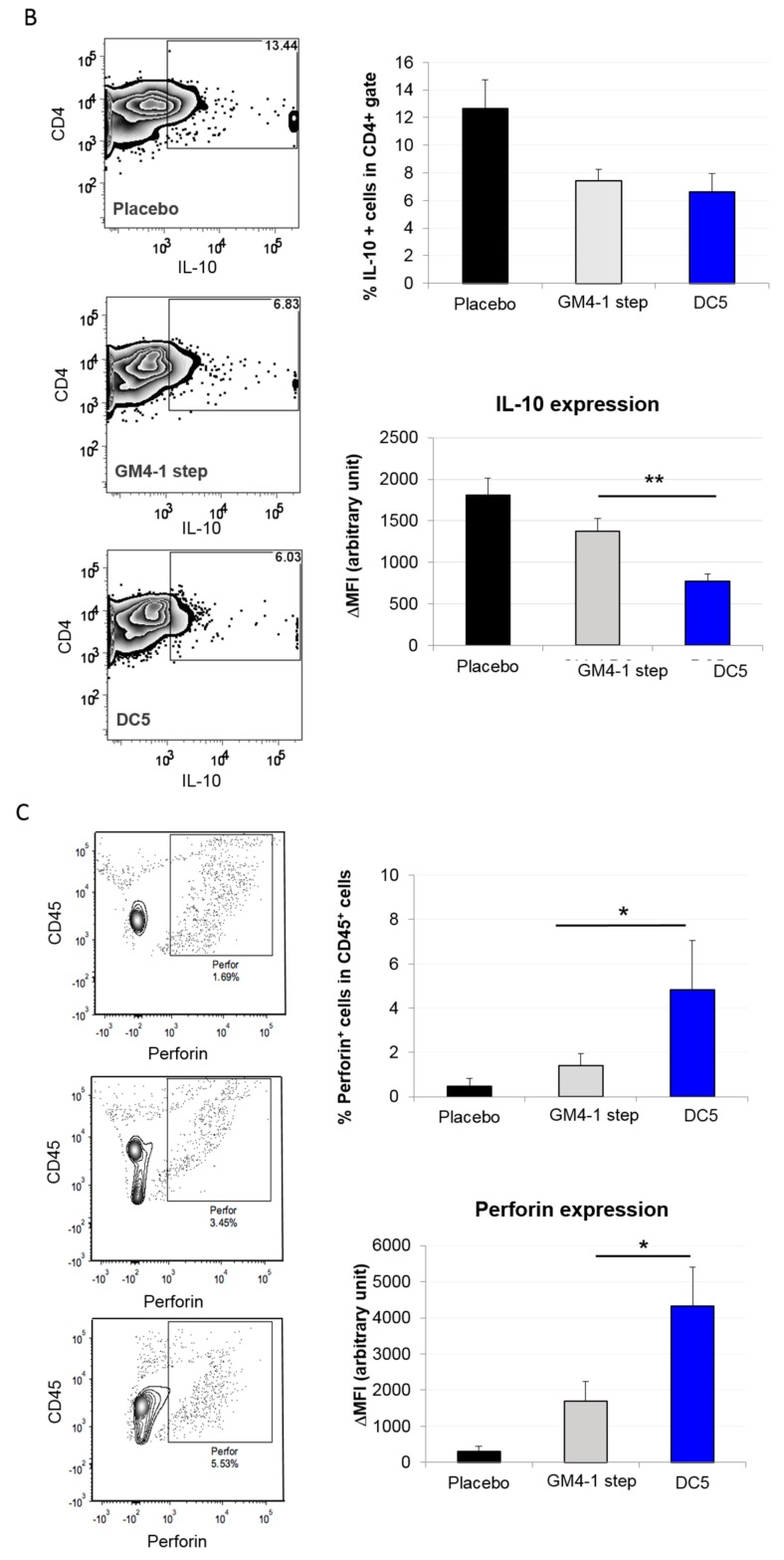

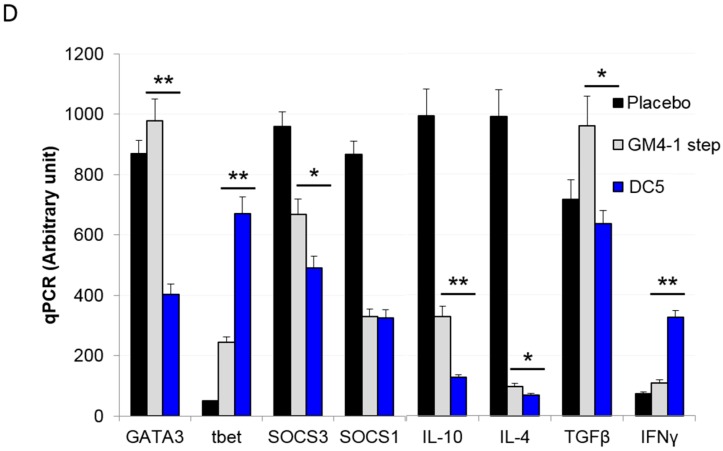
Vaccination with DC5 DCs increases cell activation and cytotoxicity of the T cell compartment at tumor site. (**A**–**C**) ID8 fast cells were injected i.p. in 8–10 weeks old female C57BL6 mice; mice were then vaccinated i.p. with the indicated DC formulation (or placebo) on day 7, day 14 and day 21 post tumor inoculation. 42 days post tumor inoculation cells isolated from the ascite fluid (in the case of placebo control animals) or obtained by peritoneal lavage (in vaccinated animals that presented no ascites) were stained with the indicated markers and analyzed by flow cytometric analysis. (**D**) mRNA levels of indicated markers were analyzed by qPCR in CD45^+^ cells isolated as reported above. Data are representative of at least 3 independent experiments. Significant differences were assessed with unpaired Student’s t test and indicated with asterisks: * *p* < 0.05; ** *p* < 0.01. (DC: dendritic cells; qPCR: quantitative polymerase chain reaction).

Based on the fact that vaccination with the DC5 formulation conferred the highest survival advantage in the present study, we decided to further characterize its induced T cell response and compare it with the one elicited by classical GM4-1 step DC vaccination which is the current golden standard for DC vaccines in clinical settings. To achieve this, we first established metastatic tumors by injecting i.p. ID8 cells (day 0) in 8–10 week old female C57BL6 mice, followed by i.p. vaccination with DCs (or placebo) on days 7, 14 and 21 post tumor inoculation. Then, at 42 days post tumor inoculation, we isolated cells from the peritoneal cavity and performed a flow cytometric analysis for different markers of cell cytotoxicity. Interestingly, we observed that in the CD8^+^ cell compartment both the expression (in terms of ΔMFI) and the percentage of granzyme B^+^ T cells were significantly higher in DC5 vaccinated animals compared to animals vaccinated with GM4-1 step DCs or placebo (Figure 7A). A similar analysis was also repeated for CD8^+^ T cells isolated by negative selection from draining lymph nodes and spleen, showing that DC5 vaccination induced significantly higher levels of granzyme B^+^ T cells also in these animal loci compared to the other two conditions (authors personal observation). Additionally, in the peritoneum cell compartment we also observed that, although the percentage of CD4^+^ T cells expressing IL-10 was comparable between animals vaccinated with GM4-1 step DCs and DC5, the amount expressed (in terms of ΔMFI) in the latter case was significantly lower (Figure 7B). Interestingly, CD45^+^ peritoneal cells form DC5 vaccinated animals also presented higher expression (in terms of ΔMFI) and percentage of perforin^+^ cells compared to control counterparts (Figure 7C).

Given that we used an intra-peritoneal model of ovarian cancer, we also performed a qPCR analysis of the whole CD45^+^ population from peritoneum of vaccinated animals to get a better understanding of the tumor microenvironment. Analyses showed that DC5 vaccinated animals were characterized by significantly lower *GATA3*, *SOCS3*, *IL-10*, *IL-4*, *TGF-β* mRNA and higher *tbet* and *IFNγ* levels compared to GM4-1 step vaccinated animals; all indicative of a Th1-skewed T cell response (Figure 7D).

**Figure 8:**
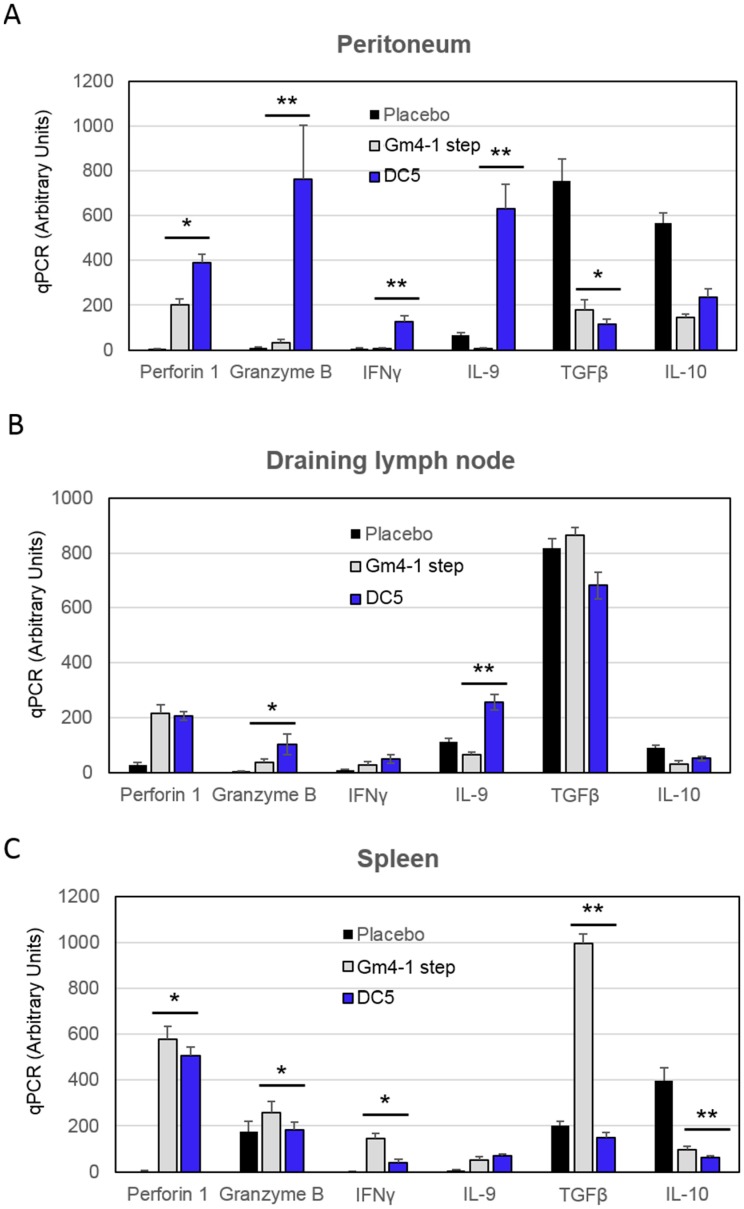
T cells isolated from DC5-vaccinated animals and stimulated ex-vivo with IL-12 present a more activated and cytotoxic mRNA profile. (**A**–**C**) T cells isolated from animals vaccinated as reported in Figure 6 were cultured for 24 h in the presence of IL-12. mRNA levels of indicated markers were then analyzed by qPCR. Data are representative of at least 3 independent experiments. Significant differences were assessed with unpaired Student’s t test and indicated with asterisks: * *p* < 0.05; ** *p* < 0.01. (IL-12: interleukin-12).

Finally, we cultured T cells isolated from vaccinated animals with autologous DCs pulsed with LSQ (or unpulsed DCs in case of placebo), in the presence of IL-12 for 24 h and analyzed cytokine levels by qPCR. T cells isolated from the peritoneum of animals vaccinated with DC5 were superior in terms of *perforin*, *granzyme B*, *IFNγ* and *IL-9* mRNA levels compared to both the respective placebo and classical GM4-1 step DC counterparts (Figure 8A). In the instance of T cells isolated from draining lymph nodes, DC5 vaccination induced higher *granzyme B* and *IL-9* mRNA levels compared to GM4-1 step vaccination; while *perforin* and *IFNγ* levels were comparable between the two conditions (Figure 8B). On the other hand, T cells isolated from the spleen of DC5 vaccinated animals showed lower *granzyme B*, *IFN-γ*, *TGF-β* and *IL-10* mRNA levels, compared to GM4-1 step vaccinated animals (Figure 8C).

These results, and in particular the pronounced *perforin 1*, *granzyme B*, *IFNγ*, and *IL-9* mRNA expression by peritoneal T cells along with low levels of *IL-10* and *TFGβ* mRNA are indicative of a high anti-tumor effector potential induced by vaccination with DC5 DCs.

## 3. Discussion

It is known that GM-CSF and IL-15 can differentiate both mouse bone marrow cells and human CD14^+^ monocytes into DCs (IL-15 DCs) [18,39]. Recently, it has been shown that these DCs can efficiently initiate both Th1 and Th17 responses [17] and mount an anti-cancer immune response against melanoma [40]. Thus, several groups subsequently brought these cells into clinical trials for cancer therapeutics with moderate success (NCT01456104, NCT01189383) [16,41]. On the other hand, DCs differentiated in the presence of GM-CSF and IL-4 (IL-4 DCs) represent the gold standard for DC therapeutic vaccination and have been tested in clinical trials for more than 20 years [42]. Despite the many efforts and advancements over the years, data meta-analysis demonstrated that this therapeutic intervention could only increase overall survival by ~20%, to date [43]. Given the crucial role played by DCs in orchestrating both innate and adaptive immune responses it is generally believed that new improvements could further improve clinical outcomes and harness the true therapeutic potential of DCs. In search for a more powerful vaccine, we compared the efficacies of IL-15 DCs and IL-4 DCs in the context of a metastatic murine ovarian cancer model. Differentiation of bone marrow cells to DCs in the presence of GM-CSF and IL-15, followed by the same maturation stimuli as conventional IL-4 DCs, resulted in higher CD86, MHC-II expression and higher IL-12p40 generation compared to the latter, indicating that this DC preparation expresses more co-stimulatory molecules and might be better equipped to activate T cell responses skewed towards a Th1 type. However, these DCs generated lower amounts of IFNγ and TNFα and similar IL-10 production to that produced by conventional GM4-1 step DCs.

The CD40/CD40L axis is an important licensing signal that enables DCs to subsequently prime naïve cytotoxic T lymphocytes [44] and CD40 ligands have also been previously shown to be potent inducers of DC maturation and activation [30,31,32]. On the other hand, it is well known that after stimulation, dendritic cells produce high levels of IL-10 [33,34], one of the most potent immunosuppressive cytokines, partially hampering DC full maturation. Previous evidence suggests that the addition of an anti-IL10 antibody blocks these IL-10 autocrine immunosuppressive effects, leading to increase DC maturation and T cell activation [33]. Hence, based on this collective evidence we then further introduced a blocking antibody against the IL10 receptor and a CD40 ligand during the first step of the maturation process in an attempt to further improve DC efficacy. In the last 24 h of the maturation process we further introduced LPS, IFNγ and CpG, three canonical maturation stimuli, to ensure full maturation status. Phenotypic analysis of the so-obtained DCs showed a drastic reduction of IDO expression, and an increase in IL-12/23p40, TLR4 and MHC-II expression, compared to classical LPS/IFNγ maturation. Interestingly, previous observations demonstrated the role of CD40L in upregulating TLR4 expression [45], suggesting that, in our case, pre-incubation of DCs with CD40L may indeed induce higher TLR4 expression leading to a higher responsiveness to subsequent LPS stimulation (a known putative TLR4 ligand), ultimately promoting a higher DC maturation status. Either way, as a consequence of their higher immunogenic phenotype, GM-15-2 step DCs elicited a T-cell response strongly skewed toward Th1 when co-cultured with splenic, lymph node or peritoneal T cells isolated from tumor bearing animals, when compared to those elicited by canonical GM4-1 step, GM4-2 step or GM15-1 step control counterparts. A corresponding significant improvement on overall survival was also observed in a mouse model of ovarian cancer upon therapeutic vaccination with GM15-2 step DCs.

Previous observations demonstrated that a strong interplay exists between malignant cells and host cells present in the tumor proximity (e.g., tumor-associated macrophages and fibroblasts, T cells, etc.) to create a tumor-promoting and immunosuppressive tumor microenvironment (TME) [46,47]. One of the major players in this context is prostaglandin E_2_ (PGE2), a lipid metabolite produced by cyclooxygenase enzymes (COX1-2) whose activity has been linked to tumor progression [48] and inversely correlated with CD8^+^ T cell tumor infiltration and patient survival [49]. Several studies demonstrated that, in addition to inhibiting T cell interleukin-2 (IL-2) production [50], proliferation [50] and tumor infiltration [49], PGE2 has also a major impact during the DC early stage development, inducing a shift towards an immunosuppressive activity [37,38], impairing DC differentiation, IL-12 production [36], DC function [37,38], promoting the development of tolerogenic DCs [38] and hence overall contributing to DC dysfunction in cancer [51]. Based on this evidence, we therefore decided to introduce PGE2 and PGE2 receptor-blocking antibodies during both DC differentiation and maturation process, in an attempt to counteract these actions [52]. Indeed, our results demonstrated that inhibiting PGE2 signaling improved DC phenotype and yielded a marginal survival advantage in vivo. In fact, the so-obtained DCs (designated as “DC5”) not only expressed higher levels of MHC-I and MHC-II, IL-12 and IL-1α compared to GM15-2 step DCs, but also elicited an in vitro strong Th1 response marked by high IFNγ and low IL-4, TGFβ and IL-10 from T cells isolated from different compartments of tumor bearing animals. While generally comparable to the GM15-2step induced ones, these responses were even improved in certain instances according to the different tested T cell compartments. Furthermore, when tested in vivo in the ID8 ovarian cancer mouse model, DC5 proved to be a much stronger therapeutic vaccine compared to GM15-2 step DCs, conferring appreciable survival advantage as well as a tumor specific memory response, as indicated by adoptive transfer experiments. T cells isolated from animals therapeutically vaccinated with DC5 DCs in fact conferred a considerable survival advantage and delayed tumor progression when transferred to naïve animals, compared to T cell transfer from placebo controls, demonstrating the tumor specificity of the DC5-induced response. Furthermore, characterization of the T cell compartment from vaccinated animals showed that DC5 vaccination is able to induce a more immunogenic and cytotoxic T cell profile. In particular, T cells in the tumor proximity (peritoneum) presented higher perforin, granzyme B, lower IL-10 and a more beneficial mRNA profile, compared to classical GM4-1 step vaccination. These data, taken collectively strongly suggest that the protocol here developed for differentiation and maturation of DC5 DCs from their bone marrow precursors constitute a valid and more beneficial alternative to standard DCs obtained with “canonical” IL-4, LPS and IFNγ stimuli. Furthermore, the work here presented was carried out with immune cells obtained from tumor-bearing mice. This aspect is of crucial importance considering previous studies reporting several important functional and numeric deficiencies of DC induced by tumors [53,54,55,56], further strengthening the evidence here presented in a more physiological and clinically relevant context.

On the other hand, despite the fact that DC5 vaccination appreciably increased the median survival against a huge metastatic tumor load, these DCs still failed to cure mice in the present study, partly due to the aggressiveness of this tumor model and partly due to the complicated tumor microenvironment which is driving the field towards combinatorial therapy. Recently, it has been proposed that the therapeutic effects of DC vaccines could be potentially further boosted with their use in combination with checkpoint blockade inhibitors such as cytotoxic T-lymphocyte-associated protein 4 (CTLA-4) and Programmed cell death protein 1 (PD-1) blocking antibodies [10]. In fact, in this way, while on one side the DC vaccine would stimulate a tumor-specific T cell response, on the other, immune checkpoint inhibitors would further sustain the clonal expansion and cytotoxicity of these DC vaccine-induced T cells. Hence, we envisage that future work should test and address this tantalizing hypothesis with improved DC formulations such as DC5 both in the mouse model and in the clinic to potentially improve therapeutic outcomes of DC vaccines, especially in the case of more advanced stage tumors.

## 4. Materials and Methods

### 4.1. Reagents

Recombinant murine GM-CSF, IL-4, IL-15 and IFNγ were purchased from Peprotech, Rocky Hill, NJ, USA. Anti-IL-10R (CD210) antibody was purchased from Biolegend, San Diego, CA, USA. Anti-CD40 monoclonal antibody (FGK-45) was procured from Enzo Lifesciences, Farmingdale, NY, USA. All fluorescent antibodies and their isotype controls were purchased from Biolegend, USA unless stated otherwise. Anti-IL-12p35-eFluor660, anti-IL-10-PE, anti-TNFα-PE/Cy7, anti-IFNγ-APC, anti-IL-12/23p40-PerCP/Cy5.5, anti-TLR9 (CD289)-biotin, anti-Thymic stromal lymphopoietin (TSLP) functional grade antibodies and Human/Murine TGFβ ELISA kit were purchased from eBiosciences, Waltham, MA, USA. Anti-TGFβ1-LAP antibody was purchased from Thermo Scientific, Waltham, MA, USA. Anti-SOCS1 and anti-SOCS3 antibodies were purchased from Millipore (Upstate), Burlington, MA, USA. Anti-Prostaglandin E2 (PGE2) and anti-prostaglandin receptor 2 (EP2) antibodies were purchased from Abcam, Cambridge, MA, USA. OptEIA ELISA sets for mouse IFNγ, IL-10 and IL-4 were from BD Biosciences, San Jose, CA, USA. ODN 1585 CpG was purchased from InvivoGen, San Diego, CA, USA. Cell culture medium and Dulbecco’s Phosphate-Buffered Saline (DPBS) were from CellGro Media Tech, Waltham, MA, USA. Fetal Calf Serum was purchased from Gibco, Life Technologies, Waltham, MA, USA. CD45^+^ cell purification system was purchased from Miltenyi Biotech, Bergisch Gladbach, Germany. Unless mentioned otherwise, all other reagents were from Sigma-Aldrich, Darmstadt, Germany. All kits for qPCR and kit for untouched T cells were purchased from Invitrogen, Waltham, MA, USA. Streptavidin-PE, anti-TLR4-APC, anti-MHC-II (IA/IE-AF647), CD86-AF488, CD83-PE, F4/80-BV421, goat anti-rat IgG-AF647 and goat anti-rat IgG-AF488 were purchased from Biolegend.

### 4.2. Cell Lines and Animals

ID8 represents a cell line derived from spontaneous malignant transformation of C57BL/6 mouse cells in vitro [57] and was a generous gift from Dr. Paul F. Terranova, University of Kansas, USA. Tumor cells were cultured in complete DMEM (Cellgro, New York, NY, USA) containing 10% heat-inactivated fetal bovine serum (FBS, Life Technologies) and antibiotics (Penstrep (Gibco, Gaithersburg, MD, USA) at 10 U/mL culture medium and normocin, (Invitrogen, Waltham, MA, USA) at 0.1 mg/mL culture medium). Cells were regularly tested for mycoplasma. Specific pathogen-free grade 6–8 week-old female C57BL/6, OT-I (C57BL/6-Tg(TcraTcrb)1100Mjb/J) and OT-II (B6.CgTg(TcraTcrb)425Cbn/J) mice were purchased from the Jackson Laboratories, Sacramento, CA, USA. Animals were maintained according to the institutional guidelines. The research obtained ethical approval under the protocol # 803648 provided by the Institutional Animal Care & Use Committee of the University of Pennsylvania (IACUC).

### 4.3. Preparation of Tumor Antigen

Squaric acid-treated tumor lysates for antigen pulsing were prepared as previously reported [14]. Briefly, ID8 cells were resuspended at a concentration of 10^8^ cells/mL in 0.06% squaric acid for 1 h at 37 °C and subsequently lysed by 6 cycles of freeze and thaw, followed by sonication (5 watt output for 15 s, 3 repeats on ice with 30 s intervals).

### 4.4. Generation of Bone Marrow-Derived Mouse DCs

DCs were generated from murine bone marrow cells, as described by Garrigan K. et al. [58] with minor modifications. Briefly, bone marrow was flushed from the long bones of ID8 tumor bearing mice. A single cell suspension was cultured in RPMI 1640 (CellGro, New York, NY, USA) supplemented with 10% heat-inactivated FBS (Life Technologies, USA), 2 mM L-glutamine (Gibco, USA), 100 U/mL penicillin (Gibco, USA), 100 µg/mL streptomycin (Gibco, USA), 10 mM HEPES pH 7.4 (Gibco, USA), 0.5 mM sodium pyruvate (Cellgro, USA), 0.5% MEM non-essential amino acids (Cellgro, USA), 0.1 mg/mL Normocin (InVivogen, San Diego, CA, USA). Cells were then differentiated according to (Figure 1) and as follows: For GM4 DCs -1 step or -2 step: with 20 ng/mL recombinant murine GM-CSF and 10 ng/mL IL-4 for 4 days (on day 2, the medium was replaced with fresh medium containing same cytokines); for GM15 1-step/2-step and DC5s: with 20 ng/mL GM-CSF and 10 ng/mL IL-15 for 4 days (on day 2, the medium was replaced with fresh medium containing same cytokines). For DC5 DCs: on day 1 with 20 ng/mL GM-CSF and 10 ng/mL IL-15; on day 2, the medium was replaced with fresh medium containing: GM-CSF (20 ng/mL), IL-15 (10 ng/mL) and both anti-PGE2 (0.1 µg/mL) and anti-EP2 Ab (0.2 µg/mL). On day 4, DCs were kept unpulsed or pulsed with squaric acid treated tumor lysate (LSQ) using ~1 tumor cell equivalent per DC and incubated for 12–16 h. After pulsing, DCs were matured as follows: for GM4-1 step and GM15-1 step: on day 5 the medium was removed and fresh medium containing 100 ng/mL LPS and 100 ng/mL IFNγ was added and incubated for 24 h at 37 °C and 5% CO_2_. For GM4-2 step and GM15-2 step: on day 4 fresh medium containing anti-CD40 Ab FGK45 (0.2 µg/mL) + anti-IL-10R blocking Ab (0.5 µg/mL) was added; on day 5, the medium was removed and fresh medium containing LPS (20 ng/mL), IFNγ (100 ng/mL) and CpG-ODN 1585 (10 μg/mL) was added and kept for 24 h. For DC5 DCs: on day 4, fresh medium containing anti CD40 Ab FGK45 (0.2 µg/mL) + anti-IL-10R blocking Ab (0.5 µg/mL) + anti-EP2 Ab (0.1 µg/mL) + anti-PGE2 (0.1 µg/mL) was added. After 3 h, the medium was removed and fresh medium containing anti-PGE2 (0.1 µg/mL), anti-EP2 Ab (0.2 µg/mL), LPS (20 ng/mL), IFNγ (100 ng/mL) and CpG-ODN 1585 (10 μg/mL) was added and incubated overnight. Cells were then harvested, stained with indicated antibodies followed by FACS analysis (Canto-II, BD bioscience, San Jose, CA, USA) or used for animal vaccination.

### 4.5. DC Vaccination

1 × 10^6^ DCs (resuspended in 500 µL DPBS) were injected i.p. on day 7, 14 and 21 following intraperitoneal inoculation of 1 × 10^6^ ID8 cells (in 500 µL DPBS) at day 0. Animals were monitored regularly for tumor growth by measuring the body weight. Mice attaining 30 g body weight were sacrificed (~40% increase in body weight over age/sex-matched normal mice).

### 4.6. Adoptive T Cell Transfer Experiments

ID8 tumor-bearing female C57BL/6 mice were vaccinated as reported above. Lymph node (LN) cells were then collected from inguinal LN on day 28 post tumor inoculation and T cells isolated by negative selection with the dynabeads mouse T cell kit (Thermofisher, Waltham, MA, USA), following manufacturer instructions. 1 × 10^6^ T cells were then injected in the tail vein of each recipient mice 3 days before tumor challenge with i.p. injection of ID8 cells (1 × 10^6^/animal). Animals were monitored regularly for tumor growth. Mice attaining 30 g body weight were sacrificed (~40% increase in body weight over age/sex-matched normal mice).

### 4.7. DC and T Cell Co-Culture

T cells from spleen or draining lymph nodes (mesenteric and inguinal) or ascites or peritoneal exudate of normal and tumor bearing (ID8) animals (receiving 3 consecutive injections of placebo (PBS) or DC vaccine on day 7, 14 and 21 post tumor inoculation) were purified using negative selection kit (Invitrogen, USA). 1 × 10^5^ T cells were plated with 1 × 10^4^ DCs and cultured for 96h in a culture volume of 200 µL. When indicated, the culture was treated with 5ng/mL recombinant mouse IL-12 (Peprotech, USA). Cells were incubated with Brefeldin A (final concentration 2 µg/mL) for 8 h and then harvested. Cells were then stained with indicated antibodies followed by FACS analysis (Canto-II, BD Biosciences) or analyzed by RTqPCR. Cell-free culture supernatants were also taken to measure levels of indicated cytokines by ELISA (OptEIA, BD Biosciences, USA) according to manufacturer procedures.

### 4.8. Reverse Transcription-Quantitative Polymerase Chain Reaction (RTqPCR)

RTqPCR was carried out using oligo-dT primer and Superscript-III reverse transcriptase (Thermo Fisher, USA). Real time PCR was performed with standard primer-probe sets obtained from Thermo Fisher, USA, following manufacturer instructions.

### 4.9. Statistical Analysis

SAS software (Version 9.3, SAS, Cary, NC, USA) and StatXact Procs 9 (Cytel, Cambridge, MA, USA) for SAS were used for statistical analysis. In particular, a two-tailed Student’s *t*-test was used to compare means of continuous measurements between two groups. The analysis of variance (ANOVA) was used to compare means among more than two groups; differences were considered statistically significant when *p* < 0.05. For animal survival, statistical analysis was performed with GraphPad Prism software using Log-rank (Mantel-Cox) test.

## 5. Conclusions

In this work, through a stepwise approach we describe a novel formulation for a DC cancer vaccine with both in vitro an in vivo improved efficacy in a mouse model, compared to canonical DC vaccine preparations that were previously available and largely in use in the field. The encouraging results here presented in the context of ovarian cancer also support future work aimed at translating this approach in the clinic, especially in combination with other adjuvant immunotherapy (such as checkpoint blockades) to further improve therapeutic outcomes.

## Supplementary Materials

The following are available online at http://www.mdpi.com/2072-6694/11/1/40/s1, Figure S1: Example of the gating strategy for DCs and for the surface marker CD86, Figure S2: DCs matured with a two-step protocol in the presence of anti-CD40 and anti-IL10R antibodies for 24 h, followed by LPS/IFNγ/CpG stimulate a more Th1-skewed T cell response compared to canonical LPS/IFNγ maturation.
