# Supplementary material for: IL-15 and a Two-Step Maturation Process Improve Bone Marrow-Derived Dendritic Cell Cancer Vaccine
Source: Cancers (Basel). 2019 Jan 4;11(1):40. doi: 10.3390/cancers11010040 (PMC6356194; doi:10.3390/cancers11010040)
Supplement: Supplementary file 1 [file cancers-11-00040-s001.pdf]

Supplementary Materials

IL-15 and a Two-Step Maturation Process Improve Bone Marrow-Derived Dendritic Cell Cancer Vaccine

Ananda Mookerjee, Michele Graciotti and Lana E. Kandalaft

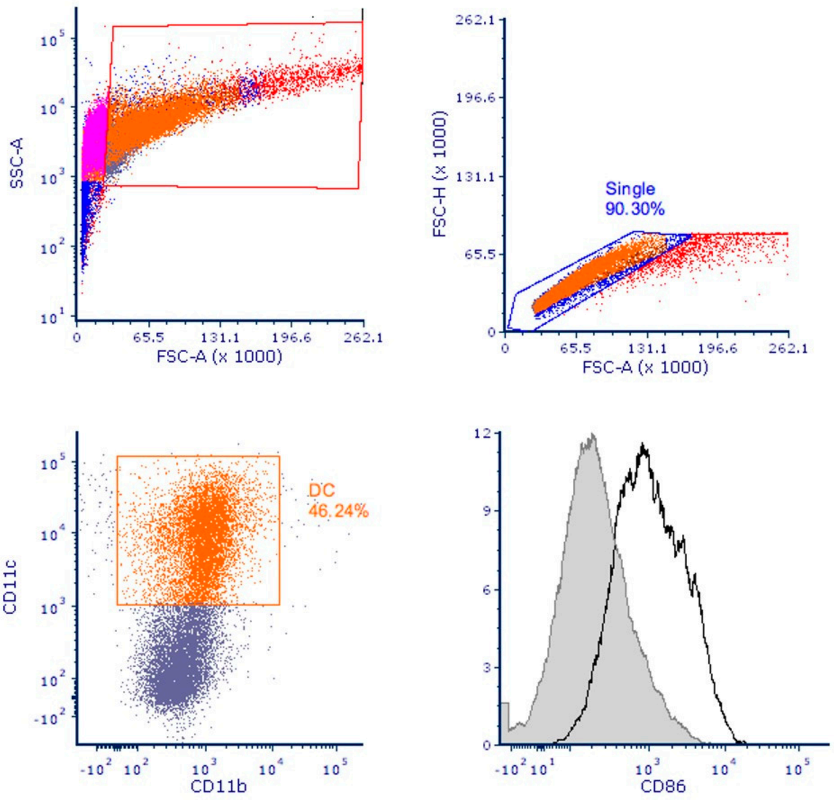

Figure S1. Example of the gating strategy for DCs and for the surface marker CD86. Cells were prepared as reported in Figure 2 (GM4-1step DCs) and analyzed by FACS; grey: isotype control staining, black: CD86 antibody staining. (DC: dendritic cells).

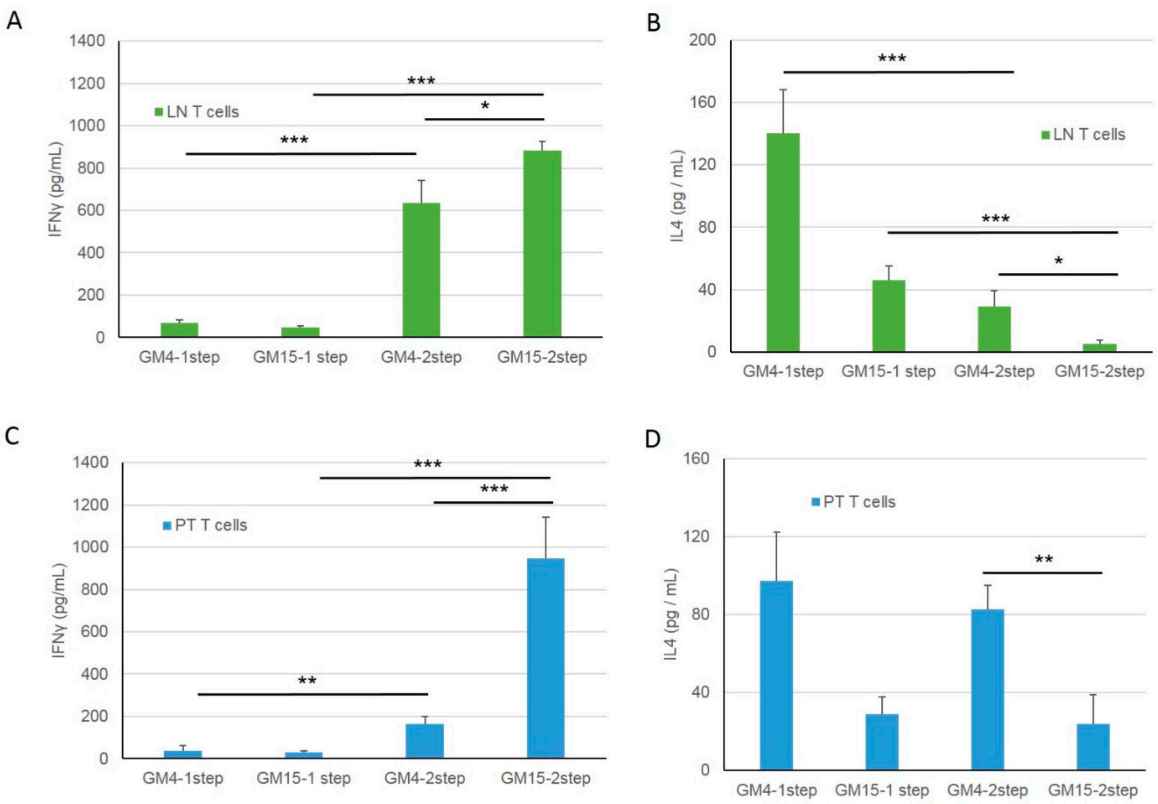

Figure S2. DCs matured with a two-step protocol in the presence of anti-CD40 and anti-IL10R antibodies for 24 h, followed by LPS/IFNγ/CpG stimulate a more Th1-skewed T cell response compared to canonical LPS/IFNγ maturation. (**A**–**D**) IFNγ and IL-4 production measure by ELISA after 24 h co-culturing of the indicated DC formulations with T-lymphocytes isolated from mesenteric and inguinal draining lymph nodes (LN) or peritoneum (PT) of tumor bearing animals. Data are representative of at least 3 independent experiments. Significant differences were assessed with unpaired Student's t test and indicated with asterisks: * $p < 0.05$; ** $p < 0.01$; *** $p < 0.005$. (CpG oligonucleotides; DC: dendritic cells; IL-10R: interleukin-10 receptor; IFN-γ: interferon-γ; LPS: lipopolysaccharide).

© 2019 by the authors. Licensee MDPI, Basel, Switzerland. This article is an open access article distributed under the terms and conditions of the Creative Commons Attribution (CC BY) license (http://creativecommons.org/licenses/by/4.0/).
